# A Novel Natural Antimicrobial Can Reduce the *in vitro* and *in vivo* Pathogenicity of T6SS Positive *Campylobacter jejuni* and *Campylobacter coli* Chicken Isolates

**DOI:** 10.3389/fmicb.2018.02139

**Published:** 2018-09-07

**Authors:** Filip Sima, Alexandros Ch. Stratakos, Patrick Ward, Mark Linton, Carmel Kelly, Laurette Pinkerton, Lavinia Stef, Ozan Gundogdu, Veronica Lazar, Nicolae Corcionivoschi

**Affiliations:** ^1^Bacteriology Branch, Veterinary Sciences Division, Agri-Food and Biosciences Institute, Belfast, United Kingdom; ^2^Department of Microbiology and Immunology, Faculty of Biology, University of Bucharest, Bucharest, Romania; ^3^Auranta, NovaUCD, Dublin, Ireland; ^4^School of Animal Science and Biotechnology, Banat University of Animal Sciences and Veterinary Medicine – King Michael I of Romania, Timisoara, Romania; ^5^London School of Hygiene and Tropical Medicine, London, United Kingdom

**Keywords:** *Campylobacter jejuni*, *Campylobacter coli*, HCT-8, attachment, invasion, gene expression

## Abstract

Human campylobacteriosis is considered one of the most common foodborne diseases worldwide with poultry identified as the main source of infection accounting for 50–80% of human cases. Highly virulent *Campylobacter* spp., positive for the Type VI secretion system (T6SS), which have an increased ability to adhere to and invade the host gastrointestinal epithelium are highly prevalent in poultry. Multidrug resistant strains of bacteria are rapidly evolving and therefore, new antimicrobials to supplement animal feed that are able to control *Campylobacter* species, are in great need. The work presented herein indicates that a novel phenolic antimicrobial, Auranta 3001, is able to reduce the adhesion and invasion of human intestinal epithelial cells (HCT-8) by two T6SS positive chicken isolates, *C. jejuni* RC039 (*p* < 0.05) and *C. coli* RC013 (*p* < 0.001). Exposure of *C. jejuni* RC039 and *C. coli RC013* to Auranta 3001 downregulated the expression of *hcp* and *cetB* genes, known to be important in the functionality of T6SS. Furthermore, the reduced adhesion and invasion is associated with a significant decrease in bacterial motility of both isolates (*p* < 0.05–*p* < 0.001) *in vitro*. Most importantly our *in vivo* results show that Auranta 3001 is able to reduce cecum colonization levels from log 8 CFU/ml to log 2 CFU/ml for *C. jejuni* RC039 and from log 7 CFU/ml to log 2 CFU/ml for *C. coli* RC013. In conclusion, this novel antimicrobial is able to reduce the pathogenic properties of T6SS campylobacters *in vitro* and also to decrease colonization *in vivo*.

## Introduction

The Gram-negative pathogen *Campylobacter* spp. is the most frequent cause of bacterial foodborne disease (EFSA and ECDC, 2017). The bacterium naturally colonizes the avian intestinal tract, where it can persist for the entire lifespan of the birds leading to contamination of poultry carcasses during slaughter, which increases the risk of human exposure to the pathogen (Shortt et al., 2016). *Campylobacter* species can cause gastro-intestinal disorders in humans, including fever, nausea, and abdominal pain. In a small group of patients, it can lead to more severe consequences such as Guillain-Barré syndrome, an acute flaccid paralysis, reactive arthritis, and inflammatory bowel disease (Chaisowwong et al., 2012; van Alphen et al., 2012; Hoseinpour et al., 2017).

In order to cause disease *Campylobacter* interacts with the gastrointestinal epithelium and colonizes the host. The full picture of the mechanisms involved is not yet known (Freitag et al., 2017), but initial progress has been made to elucidate these mechanisms (Young et al., 2007). Blocking these initial stages of infection including adhesion, motility, and chemotaxis is essential as they represent key factors for a successful infection (Morooka et al., 1985; Nachamkin et al., 1993; Yao et al., 1997; Hendrixson and DiRita, 2004; Backert and Hofreuter, 2013). More specifically these virulence factors include motility systems (*flaA* and *flaB*), adhesion to fibronectin F (*cadF*), chemotaxis (*cetB*), invasion proteins, and cytolethal distending toxins (*cdtB*), whose production causes progressive cellular distension and cell death leading to enteritis. Therefore, reducing the attachment and invasion of *Campylobacter* sp. on intestinal epithelial cells and reducing production of virulence factors such as adhesins and decreasing motility could potentially control campylobacteriosis. Besides other virulence factors, capsular polysaccharides contribute to antimicrobial resistance (Hendrixson et al., 2001; Guerry et al., 2012).

Treating *Campylobacter* infections in humans with antibiotics (e.g., erythromycin, clarithromycin, ciprofloxacin, levofloxacin, moxifloxacin) is common practice. The increasing resistance of bacteria to conventional chemicals and drugs, the decline in new antibiotic discovery over the last few decades, as well as consumer demands for natural food preservatives have encouraged research for the identification of novel natural antimicrobials (Kovac et al., 2015; Oh and Jeon, 2015a; Stratakos et al., 2018). Plant extracts have been used for decades not only as flavor enhancers, but also to extend the shelf life and microbiological safety of food (Hintz et al., 2015). Their applicability was also proven for the treatment of a range of human and animal diseases, improving human health (Holley and Patel, 2005; Teichmann et al., 2016).

Controlling *Campylobacter* spp. is considered a public health priority. Antimicrobial products are used in general with the aim of bacterial killing but this may have a damaging effect on the host microbiome. Therefore, investigations into the possibility of achieving a reduced virulence rather than a lethal effect would be of interest. Auranta 3001 has been shown to be involved in reduced virulence of *Cryptosporidium hominis* and *Cryptosporidium parvum* by downregulating CpSUB1 gene expression (Stratakos et al., 2017). In order to avoid any effect on bacterial survival and growth, and therefore apply less selective pressure for the development of resistance, sub-inhibitory concentrations of antimicrobials need to be identified. The use of sub-inhibitory concentrations is necessary for the sound investigation of the anti-virulence capacity of antimicrobials/compounds (Kummerer, 2004). Our study aimed to investigate if sub-inhibitory concentrations of a mixture of organic acids and plant extracts (Auranta 3001) can reduce the virulence of T6SS positive *C. jejuni* and *C. coli* isolates in human HCT-8 cell models and colonization of caeca in artificially infected chicken broilers.

## Materials and Methods

### Strains and Culture Conditions

*C. jejuni* RC039 and *C. coli* RC013 strains were obtained from the AFBI laboratory collection and were grown on Blood Agar Base No. 2 (Oxoid Ltd., United Kingdom) supplemented with 5% (vol/vol) defibrinated horse blood (Aquilant Scientific N.I.). The strains were grown under microaerophilic conditions at 41.5°C in 85% N_2_, 5% O_2_, and 10% CO_2_ in a Don Whitley MACS-VA500 microaerophilic workstation (Davidson & Hardy Ltd., United Kingdom) for 48 h. To enumerate viable microorganisms, suitable 10-fold dilutions were made in Maximum Recovery Diluent (Oxoid Ltd., United Kingdom). One hundred microliters of each of the 10-fold dilutions were spread on *Campylobacter* Blood-Free Selective Agar Base (Modified CCDA – Preston; Oxoid Ltd., United Kingdom) without any supplement, and plates were incubated under microaerophilic conditions at 41.5°C for 48 h.

### Chemicals

The novel antimicrobial (Auranta 3001) was supplied by Auranta – Envirotech Innovative Products Ltd and contains lactic and citric acid. The antimicrobial also contains: glycerine-based emulsifying agent, sodium chloride, sodium hydroxide, citrus extract (6%), oregano extract (1%), grape seed extract (2%).

### Infection

The ECACC human ileocecal adenocarcinoma cells (HCT-8) were grown in RPMI 1640 (Lonza, Analab Ltd., United Kingdom) supplemented with 10% fetal bovine serum and 2 mL glutamine. The cells were routinely grown in 75 cm^2^ tissue culture flasks (Sigma-Aldrich, Arklow, Ireland, SIAL0641) in a humidified incubator at 37°C with 5% CO_2_. The gentamicin protection assay was used to test the role of Auranta 3001 in the ability of *C. jejuni* RC039 and *C. coli* RC013 to adhere to and invade host epithelial cells as previously described (Corcionivoschi et al., 2009). HCT-8 cells were grown (80–90% confluence) for 22 to 24 h in six-well tissue culture plates at a concentration of 5.5 × 10^5^ cells per well. For some experiments, HCT-8 monolayers were preincubated with 0.1 and 0.5% Auranta 3001 for 1–3 h. Plate grown *C. jejuni* RC039 and *C. coli* RC013 were harvested, washed, and re-suspended in tissue culture medium at an OD_600_ of 0.3 and 0.25, respectively. The bacterial isolates were preincubated for 1–3 h in the presence of 0.1 and 0.5% Auranta 3001. The HCT-8 cells were washed with fresh culture media containing 10% FBS, and 2 ml of fresh culture medium was added to each well. Bacteria were added to give a multiplicity of infection of 100. Tissue culture plates were centrifuged at 250 × *g* for 5 min and incubated for 3 h at 41.5°C in 85% N_2_, 5% O_2_, and 10% CO_2_. To quantify the number of cell-associated bacteria, infected monolayers were washed three times with PBS and treated with 0.1% Triton X-100 in PBS at 41.5°C for 15 min. Tenfold dilutions of each well were plated onto CCDA agar and colonies enumerated after 2 days of incubation at 41.5°C in 85% N_2_, 5% O_2_, and 10% CO_2_. To quantify the number of bacteria that invaded HCT-8 cells, the infected monolayers were washed with tissue culture medium. Fresh medium (2 ml) containing gentamicin (400 μg/ml) was added to kill bacteria that were not internalized. Medium without gentamicin was added to quantify the number of bacteria that adhered to the epithelial cells. The tissue culture plates were then incubated for a further 3 h at 41.5°C and washed with fresh RPMI 1650 + 10% FBS. HCT-8 cells were lysed by the addition of 1 ml of 0.1% Triton X-100 in PBS and incubated for 15 min at 41.5°C (Corcionivoschi et al., 2009). Because in our study, we have used chicken isolates the incubation temperature was increased from 37 to 41.5°C. Tenfold dilution of the contents of each well was plated onto CCDA agar, and colonies were enumerated after 2 days of incubation. Invasion efficiency was calculated as the percentage of the total number of CFU/total initial inoculum. All assays were conducted in triplicate and repeated independently three times. The significance of differences in adhesion and invasion between samples was determined using the Student’s *t*-test. A *P*-value of <0.05 was defined as significant.

### Susceptibility to Auranta 3001

The twofold tube dilution method was used to determine the lowest concentration of Auranta 3001 that can inhibit growth of bacteria (MIC) and the lowest concentration that results in bacterial death (MBC) according to Zhu et al. (2016). Auranta 3001 was diluted (8% down to 0.0078% v/v) in Müeller Hinton broth (MHB) and thoroughly vortexed. Individual overnight bacterial cultures were harvested by centrifugation, washed with PBS, and diluted to approximately 1 × 10^6^ CFU/mL in MHB. Each tube was inoculated with approximately 5 × 10^5^ CFU/mL of this bacterial culture (final concentration). Noninoculated tubes containing the same growth medium were used as negative controls and tubes inoculated with individual bacterial cultures in MHB without Auranta 3001 were used as positive controls. Subsequently, the tubes were incubated at 41.5°C for 48 h. Tubes without visible growth were considered as below the MIC. One hundred milliliters were taken from the tubes that showed no growth and inoculated onto MHA plates, the highest dilution with no microbial growth was considered as the MBC. Each assay was repeated three times for each strain. In order to determine the sub-inhibitory concentrations used, the two pathogens were exposed to different concentrations of the antimicrobial. The concentrations that showed no effect on survival and no growth inhibition (same growth kinetics as the control) were used for the subsequent experiments.

### Motility Assay

The motility of *C. jejuni* RC039 and *C. coli* RC013 was measured after the two strains were exposed to Auranta 3001 for 1, 2, and 3 h at a concentration of 0.1 and 0.5%. In short, 5 μl of culture (grown on blood agar for 48 h and recovered in 1 ml brain heart infusion – BHI – broth) was inoculated into the center of a 20 ml semi-solid BHI plate (0.4% agar). The radius of the zone of visible growth was measured after 48 h of incubation under microaerophilic conditions at 41.5°C. The experiment was carried out in triplicate, on three different days. The results are expressed as percentage decrease compared to the control.

### Capsule Polysaccharide (CPS) Detection

Capsule polysaccharide was prepared from bacteria co-cultured with HCT-8 cells which were pretreated with Auranta 3001 and from bacteria directly exposed to Auranta 3001 following a previously described protocol (Hitchcock, 1983). Bacteria were harvested by centrifugation and suspended in 100 μl of lysis buffer containing 31.25 mM Tris-HCl (pH 6.8), 4% sodium dodecyl sulfate, 0.025% bromophenol blue, and 20% glycerol. After heating to 100°C for 5 min, 5 μl of 20 mg/ml proteinase K was added to the solution, and the tubes were incubated for 1 h at 50°C. The samples were separated on NuPageNovex 12% bis-Tris gels (Invitrogen, United Kingdom). Following electrophoresis, gels were stained with an Alcian blue (Sigma Aldrich, United Kingdom) solution containing 0.1% Alcian blue, 40% ethanol, and 5% acetic acid (Karlyshev and Wren, 2001).

### RNA Extraction and qRT-PCR

Total RNA was isolated from bacteria exposed to Auranta 3001 at a concentration of 0.1 and 0.5% for 1, 2, and 3 h by using the RNeasy^®^Plus Mini Kit (Qiagen, United Kingdom). The RNA was reverse transcribed using Transcriptor First Strand cDNA Synthesis Kit (Roche, United Kingdom) according to the manufacturer’s protocol. The mRNA levels were determined by quantitative RT-PCR using QuantiNovaSYBR^®^ Green PCR Kit (Qiagen, United Kingdom) on a LightCycler^®^ 96 (Roche, United Kingdom). The primers used (Invitrogen, United Kingdom) are described in **Table 1**, and the conditions for genes *rRNA* 16S consisted of incubating for 10 min at 95°C followed by 45 cycles of 95°C for 10 s, 55°C for 30 s, and 72°C for 10 s. A total of 5 μl of SYBR Green master mixture was used in each reaction along with 0.5 μl of 10 μM primer mixture, 3 μl of molecular grade water, and 1 μl of DNA sample. For *cetB* (10 min at 95°C, followed by 45 cycles of 95°C for 10 s, 54°C for 20 s, and a final extension at 72°C for 5 min) a total of 5 μl of SYBR Green master mixture was used in each reaction along with 1.4 μl of 20 μM primer mixture, 5.2 μl of molecular grade water, and 2 μl of DNA sample. For *hcp* (2 min at 95°C, followed by 40 cycles of 95°C for 5 s, 60°C for 10 s, and a final extension at 72°C for 5 min), a total of 5 μl of SYBR Green master mixture was used in each reaction along with 0.8 μl of 20 μM primer mixture, 7.4 μl of molecular grade water, and 1 μl of DNA sample. Relative quantity of the mRNA was calculated using the ΔCt method. *rARN 16S* gene was used as an endogenous control since it was transcribed in equal rates in both treated and untreated cells.

**Table 1:** List of primer sequences used for qRT-PCR for *C. jejuni* RC039 and *C. coli* RC013 genes associated with virulence.

Gene	Primer sequence – forward	Primer sequence – reverse	Reference
*cetB*	5′ GCCTTGTTGCTGTTCTGCTC 3′	5′ TTCCGTTCGTCGTATGCCAA 3′	Upadhyay et al., 2017
*hcp*	5′ CAAGCGGTGCATCTACTCAA 3′	5′ TAAGCTTTGCCCTCTCTCCA 3′	Harrison et al., 2014
*rRNA 16S*	5′ ATCTAATGGCTTAACCATTAAAC 3′	5′ GGACGGTAACTAGTTTAGTATT 3′	Douglas Inglis and Kalischuk, 2003

### *In vivo* Infection Assay

Twenty male chicken broilers (Ross 308) were housed in isolation units on wood shaving bedding. The temperature in the isolation unit was kept between 22 and 25°C and thermostatically controlled. Broilers were fed *ad libitum* with a standard diet as described in **Table 2**. *C. jejuni* RC039 and *C. coli* RC013 were grown as described above and suspended in sterile distilled water at a concentration of 1 × 10^7^ CFU/ml. At the age of 15 days, 10 broilers were inoculated with 0.1 ml of *C. jejuni* RC039 and the other 10 broilers with 0.1 ml *C. coli* RC013. The infected broilers were kept in separated and sterile isolation units. Five chickens from each infected group received for 3 days, *ad libitum*, water containing 0.5% Auranta 3001 with the remaining ones receiving water only. After 3 days of treatment, broilers were euthanized, and *Campylobacter* was enumerated by analyzing the cecum content. All broilers were confirmed as being *Campylobacter* free at the time of infection, using cloacal swabs. These experiments were performed in triplicate on three separate occasions. The experiments were performed according to the legislation in place (Law 471/2002 and government ordinance 437/2002) and under the supervision of National Sanitary Veterinary Agency. The ethics committee of Banat University of Agricultural Sciences and Veterinary Medicine – King Michael I of Romania, approved this work

**Table 2:** Chemical composition of basal diet.

Item	Starter	Grower	Finisher
	0–10 days	11–24 days	25–35 days
Wheat	54.623	57.553	61.300
Full fat soya	12.000	12.000	12.000
Brazilian GM hipro	25.000	21.000	17.000
Lime bulk	0.717	0.700	0.500
DCP bulk (18.1% p)	1.654	2.000	2.150
Salt bulk	0.200	0.200	0.200
Sod.bi-carbonate	0.199	0.166	0.162
DL methionine	0.487	0.435	0.378
L-lysine	0.373	0.318	0.281
Threonine	0.247	0.128	0.029
Vitamin+mineral premix	0.500	0.500	0.500
Soyabean oil	4.000	5.000	5.500
**Calculated composition (%)**			
ME Kcal/kg	2999	3081	3133.8
CP	23.12	21.53	20.04
Lys	1.45	1.308	1.17
Met+Cys	1.089	0.996	0.91
Ca	0.97	0.906	0.85
AvP	0.49	0.41	0.409

### Transepithelial Resistance of Cellular Tight Junctions (TEER)

Transepithelial resistance measures the integrity of cellular tight junctions and is a suitable method to be used in cell culture monolayers for the purpose of measuring the effect of bacteria during infection *in vitro* (Srinivasan et al., 2015). This methodology measures the electrical resistance, in ohms, as a measure of cellular barrier integrity. For the purpose of TEER measurement, the HCT-8 cells were grown on 0.4 μm and 12 mm pore size transwell inserts (Corning) and selected based on the formation of a confluent monolayer. Our aim was to investigate the effect of Auranta 3001 on the barrier properties of HCT-8 cells by taking TEER measurements at 3 h postinfection (±0.1 and 0.5% Auranta 3001) using an EVOM X meter connected to an Endohm chamber (World Precision Instruments).

### Statistical Analysis

All experiments were performed in triplicate and data presented as mean ± SEM. To measure the statistical significance of the infection assays and gene expressions results, we used two tailed Student’s *t*-test. A group difference was assumed to be statistically significant when *P*? < ?0.05. All results were expressed as means ± SD.

## Results

### Identification of the Sub-Inhibitory Concentrations

In order to investigate if the antimicrobial agent has any effect in reducing pathogenicity, by attenuating bacterial virulence factors, we first had to identify the sub-inhibitory concentrations for both isolates (*C. jejuni* RC039 and *C. coli* RC013). Strong antimicrobial activities against both *C. jejuni* RC039 and *C. coli* RC013 were observed. The minimum inhibitory concentrations were 1% for *C. jejuni* RC039 and 2% for *C. coli* RC013, and minimum bactericidal concentrations were recorded at similar concentrations (**Table 3**). We have concluded that concentrations of 0.1 and 0.5% were most appropriate for our next investigations in order to avoid any antimicrobial killing or any effect on bacterial growth

**Table 3:** Minimum inhibitory concentration (MIC) and minimum bactericidal concentration (MBC) activity of the Auranta 3001.

Strains	Origin	MIC	MBC
*Campylobacter jejuni* RC039	poultry	1%	1%
*Campylobacter coli* RC013	poultry	2%	2%

*The antimicrobial was tested at concentrations from 8 to 0.0078% (v/v) in three independent experiments*.

### Auranta 3001 Reduces the Motility of *C. jejuni* RC039 and *C. coli* RC013

**FIGURE 1:**
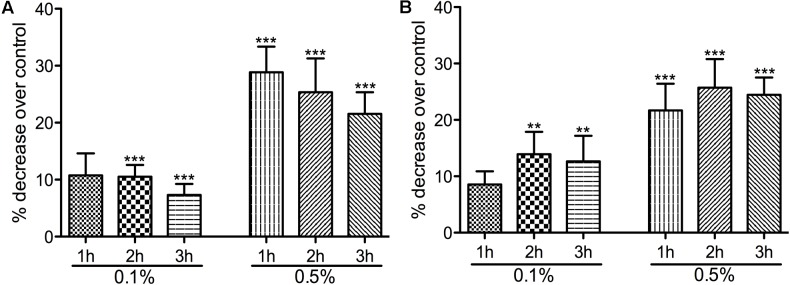
The effect of Auranta 3001 on *C. jejuni* RC039 and *C. coli* RC013 motility. **(A)** shows the percentage decrease of *C. jejuni* RC039 motility and **(B)** of *C. coli* RC013 over control. *Asterisks* indicate significant differences (^∗^*p* < 0.05; ^∗∗^*p* < 0.01; ^∗∗∗^*p* < 0.001). *Error bars* represent the standard deviation of means from three different experiments, each containing triplicate samples.

Having identified the sub-inhibitory concentrations, we have next studied the potential impact on virulence by the reduction of bacterial motility (**Figure 1**). Motility plate assays showed that both strains were highly motile but the pretreatment with 0.1 and 0.5% Auranta 3001 significantly reduced motility in a dose and time dependent manner. As reflected in **Figures 1A,B**, a concentration of 0.1% Auranta 3001 significantly reduced motility after only 2 h for both *C. jejuni* RC039 (*p* < 0.01) and *C. coli* RC013 (*p* < 0.05). A concentration of 0.5% Auranta 3001 reduced bacterial motility by more than 20% (*p* < 0.001) after 1, 2, and 3 h for *C. jejuni* RC039 (**Figure 1A**), and for *C. coli* RC013 (**Figure 1B**) when compared to the control. These results suggested that this antimicrobial can have a negative impact on the motility of *C. jejuni* and *C. coli* T6SS positive isolates.

### Auranta 3001 Reduces *C. jejuni* RC039 and *C. coli* RC013 Virulence *in vitro* and Decreases Colonization *in vivo*

**FIGURE 2:**
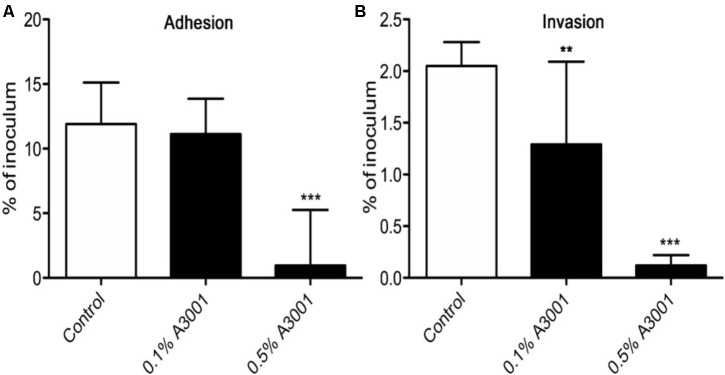
Adhesion and invasion of HCT-8 cells by *C. jejuni* RC039. The adherence **(A)** and invasion **(B)** of HCT-8 cells in the presence of Auranta 3001. Results are expressed as percentages of the initial inoculum. *Asterisks* indicate significant differences (Student’s *t*-test ^∗^*p* < 0.05; ^∗∗^*p* < 0.01; ^∗∗∗^*p* < 0.001). *Error bars* represent the standard deviation of means from three different experiments, each containing triplicate samples.

**FIGURE 3:**
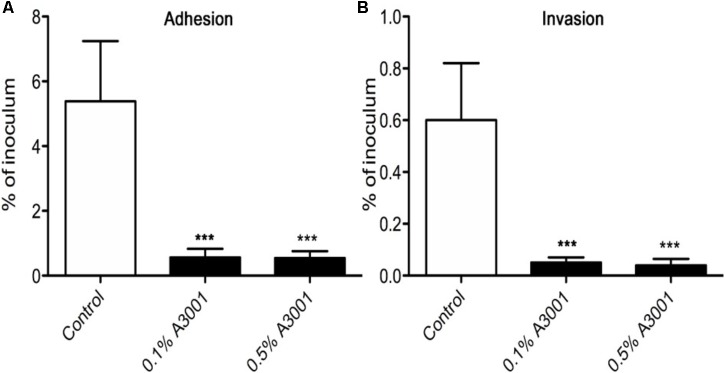
Adhesion and invasion of HCT-8 cells by *C. coli* RC013. The adherence **(A)** and invasion **(B)** of HCT-8 cells in the presence of Auranta 3001. Results are expressed as percentages of the initial inoculum. *Asterisks* indicate significant differences (Student’s *t*-test ^∗^*p* < 0.05; ^∗∗^*p* < 0.01; ^∗∗∗^*p* < 0.001). *Error bars* represent the standard deviation of means from three different experiments, each containing triplicate samples.

To demonstrate if the negative effect observed on motility also translates to reduced virulence we performed *in vitro* infection assays as described in Materials and Methods section. The presence of Auranta 3001 in the culture media throughout the infection assay significantly reduced the adherence of *C. jejuni* RC039 to HCT-8 cells (**Figure 2A**) at a concentration of 0.5%, while the negative effect on invasion (**Figure 2B**) was significant at both concentrations (*p* < 0.001). In the case of *C. coli* RC013, the negative effect on adherence (**Figure 3A**) and invasion (**Figure 3B**) was significant (*p* < 0.001) at both 0.1 and 0.5% Auranta 3001. The invasion ability of *C. jejuni* RC039 and *C. coli* RC013 was significantly diminished (*p* < 0.001) when HCT-8 cells only were exposed to Auranta 3001 suggesting that host related infection pathways are affected by the pretreatment (**Supplementary Figure 1**). Moreover, if the bacteria only are pretreated, prior to infection their invasion capacity is also dramatically reduced (**Supplementary Figure 2**). Overall, these data indicate that the reduced infection could be due to biological changes in both the host and the bacterium. The successful reduction in virulence obtained *in vitro* was extremely encouraging and as a consequence we then investigated the effect of Auranta 3001 in preventing cecum colonization of artificially infected chicken broilers. *In vivo*, quantification of viable bacteria in cecum content showed significant decrease (*p* < 0.05) in the ability of *Campylobacter* to colonize the gastrointestinal tract. For *C. jejuni* RC039, the decrease was from log 8 CFU/ml to 2 log CFU/ml and for *C. coli* RC013 from 7 log CFU/ml to 2 log CFU/ml (**Table 4**). In conclusion, the addition of Auranta 3001 to the drinking water significantly reduced the ability of *C. jejuni* RC039 and *C. coli* RC013 to colonize the broiler cecum when compared to controls.

**Table 4:** Detection of viable campylobacters (log_10_ CFU/ml).

Experiment	*C. jejuni* RC039		*C. coli* RC013	
	+A3001	-A3001	+A3001	-A3001
A	3.9 × 10^2a^	7.3 × 10^8^	4.2 × 10^2a^	5.1 × 10^6^
B	2.7 × 10^2a^	8.1 × 10^9^	2.7 × 10^3a^	3.3 × 10^7^
C	1.2 × 10^2a^	6.6 × 10^8^	3.9 × 10^2a^	4.7 × 10^6^

*^a^Significantly (*P* < 0.05) less than the untreated control.*

*A, B, and C represent three replicate experiments performed in three separate occasions*.

### Changes in Capsule Polysaccharide (CPS) Profiles of *C. jejuni* RC039 and *C. coli* RC013 Following Exposure to Auranta 3001

**FIGURE 4:**
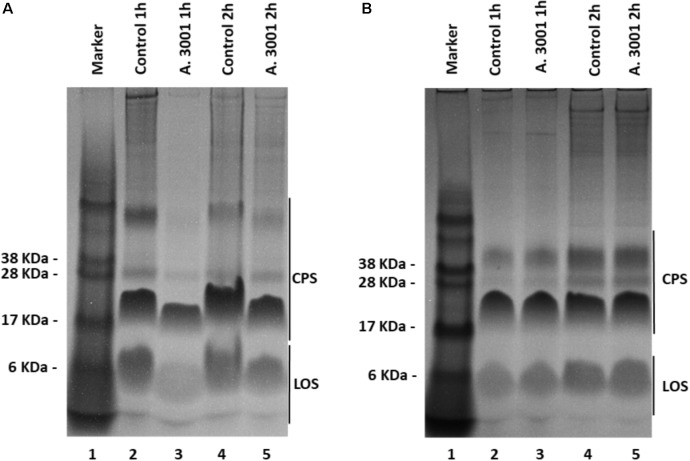
Alcian blue profiles of *Campylobacter jejuni* RC039 **(A)** and *Campylobacter coli* RC013 **(B)** capsule polysaccharides when co-cultured with HCT-8 cells pretreated with 0.1% Auranta 3001.

Having observed that exposure of bacteria to the antimicrobial reduces their virulence we then investigated the production of CPS, a major virulence and colonization factor in campylobacters. Our results show that exposure of HCT-8 cells to 0.1% Auranta 3001 prior to infection leads to a significant decrease in the amounts of CPS detected on co-cultured *C. jejuni* RC039 (**Figure 4A**, lanes 3 and 5) compared to control. Interestingly a similar effect was not detected for *C. coli* RC013 in which case no difference was observed when compared to control (**Figure 4B**).

### Auranta 3001 Downregulates *hcp* and *cetB* Gene Expression in *C. jejuni* RC039 and *C. coli* RC013

**FIGURE 5:**
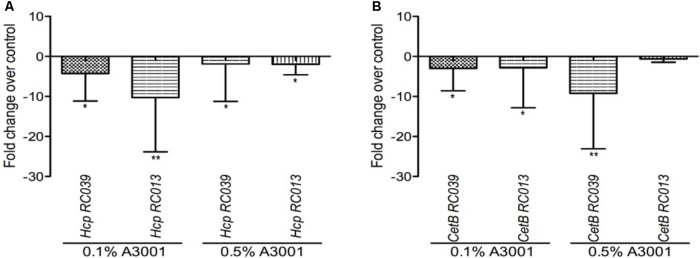
Effect of Auranta 3001 on *C. jejuni* RC039 and *C. coli* RC013 *cetB* **(B)** and *hcp* gene **(A)** expression after 3 h of exposure to 0.1 and 0.5% Auranta 3001. Asterisks indicate significant differences (Student’s *t*-test ^∗^*p* < 0.05; ^∗∗^*p* < 0.01). *Error bars* represent the standard deviation of means from three different experiments.

The effect of Auranta 3001 on the expression of *C. jejuni* RC039 and *C. coli* RC013 *hcp* gene is shown in **Figure 5**. The qRT-PCR results revealed that the antimicrobial agent reduced the transcription levels of *hcp* and the energy chemotaxis related gene (*cetB*). Exposure of *C. jejuni* RC039 to 0.1% Auranta 3001 (**Figure 5A**) resulted in a fivefold reduction of *hcp* gene expression with a 10-fold reduction in *C. coli* RC013 (*p* < 0.01). Interestingly only twofold downregulation of the *hcp* gene (**Figure 5A**) was observed when both isolates were exposed to 0.5% Auranta 3001. In the case of *cetB* gene expression in *C. jejuni* RC039, we show a significant marked increase (*p* < 0.01) in downregulation following exposure from fivefold at 0.1% to 10-fold at 0.5% (**Figure 5B**). For *C. coli* RC013, the downregulation of *cetB* was more significant following exposure to 0.1% Auranta 3001 compared to 0.5% (*p* < 0.05).

### Auranta 3001 Prevents Tight Junction Disruption During Infection

**FIGURE 6:**
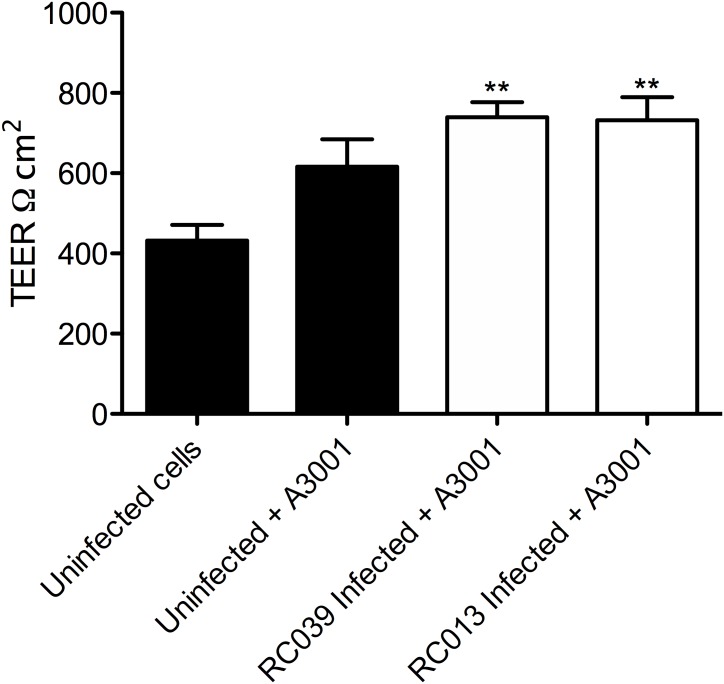
Transepithelial resistance of HCT-8 cells infected with *C. jejuni* RC039 and *C. coli* RC013 at 3 h postinfection using 0.5% Auranta during infection. Asterisk indicates significant difference (^∗∗^*P* < 0.001) compared to uninfected cells. Error bars indicate standard deviations.

As shown in **Figures 2**, **3** Auranta 3001 reduces the virulence of both *C. jejuni* RC039 and *C. coli* RC013 when used at concentrations between 0.1 and 0.5%. We have hypothesized that the effect could be caused by an increase in tight junction resistance. In order to test our hypothesis, TEER was measured at 3 h postinfection in the presence of 0.1 and 0.5% Auranta 3001. By 3 h postinfection, there was a significant increase (*p* < 0.01) in TEER in both uninfected and infected cells when Auranta 3001 was present in the infection media (**Figure 6**). The results were similar when 0.1% Auranta 3001 was used (data not shown). These results indicate that Auranta 3001 inhibits the disruption of the tight junctions during infection preventing pathogen translocation and subsequent infection.

## Discussion

*Campylobacter* remains an important microbiological contaminant of chicken products despite substantial efforts to reduce the infection (Hermans et al., 2011a). An effective preharvest control strategy that reduces pathogen colonization in the cecal environment could reduce the risk of fecal shedding and therefore product contamination considering that *Campylobacter* resides primarily in the cecal crypts of birds (Beery et al., 1988). Administration of antimicrobial treatment through feed can be applied as a practical method for controlling pathogen colonization. Plant-derived compounds or phytochemicals represent a large unexploited resource that can serve as a safe and effective alternative for controlling pathogens in birds, considering that there is an increasing number of customers that demand antibiotic free chickens. In addition, in the EU the use of antibiotics as growth promotors is not permitted (Hermans et al., 2011b; Wagle et al., 2017).

Bacterial virulence factors such as adhesion are important for colonization; a reduction in this particular attribute could minimize *Campylobacter* ability to attach to the epithelial cells, hence its colonization of poultry (Jin et al., 2001; Hermans et al., 2011a). Previously, Byrne et al. (2007) showed that *Campylobacter* spp. attaches and invades both primary chicken enterocytes and human epithelial cells with similar efficiency. The effect of other natural antimicrobials has been widely investigated in different studies of *Campylobacter* spp. infection. Blackberry (*Rubus fruticosus*) and blueberry (*Vaccinium corymbosum*) extracts have been shown to significantly reduce the adhesion and invasion to epithelial cells (Salaheen et al., 2014). Other extracts from *Artemisia ludoviciana, Acacia farnesiana, Cynarascolymu* ssp., *Opuntiaficus indica* (Castillo et al., 2014), as well as β-resorcylic acid, have shown similar effects (Wagle et al., 2017). During the course of this study, we have explored the efficacy of Auranta 3001 (a mixture of organic acids and plant extracts) in reducing the ability of *C. jejuni* and *C. coli*, T6SS positive isolates, to invade epithelial cells *in vitro*. Our results indicate that the antimicrobial agent has a direct effect on the pathogen as following pretreatment of *C. jejuni* RC039 and *C. coli* RC013 a statistically significant reduction in invasiveness was observed (*p* < 0.001). This effect was slightly more pronounced, compared to control, with the increased exposure time and the concentration of Auranta 3001. Moreover, we have observed similar results when Auranta 3001 was used to pretreat the HCT-8 cells before infection, suggesting that the antimicrobial agent may potentially interfere with the host cell metabolic pathways, which subsequently affect the ability of bacteria to infect. This theory is supported by a study, which indicates that carvacrol, the main component of oregano, does not inhibit bacterial growth but significantly reduces the virulence potential of *C. jejuni* and protects against cellular infection of INT-407 cells (van Alphen et al., 2012). Similarly, Citral, which is found in citrus extracts, is able to significantly suppress the attachment and invasion to Caco-2 cells and inhibit the expression of genes involved in the attachment and invasion of host cells by *C. sakazakii* (Shi et al., 2017).

Cellular tight junctions are a physical barrier against pathogen intrusion and strengthening them is key in the development of novel strategies against *Campylobacter* spp., infection (Hatayama et al., 2018). *Campylobacter* ability to disrupt the tight junctions and invade via the basolateral site of eukaryotic cells has been reported in several studies (Monteville and Konkel, 2002; Chen et al., 2006). The increase in TEER, observed in our studies, suggests that the tight junctions of infected cells are strengthened in the presence of Auranta 3001, which could have contributed to the reduction in adhesion and invasion of the two isolates.

To investigate the potential mechanism of action of Auranta 3001, we evaluated the effect of the antimicrobial on various virulence attributes of *C. jejuni* and *C. coli*. Motility is crucial to *Campylobacter* pathogenesis both *in vitro* and *in vivo*, and it has been shown that diminished motility results in reduced ability to invade epithelial cells *in vitro* (Yao et al., 1994; Golden and Acheson, 2002; Stahl et al., 2014). Our study shows that the antimicrobial agent significantly reduces the motility of *C. jejuni* RC039 and *C. coli* RC013 in a time and dose-dependent manner. Similar results were reported in a recent study, which describes that preexposure of *Campylobacter* spp. to different concentrations of organic oils, such as carvacrol, eugenol, and thyme reduced the motility and invasion of *C. jejuni* into intestinal epithelial cells, without affecting the normal function of the cells (Upadhyay et al., 2017). A citric-based disinfectant applied at sub-inhibitory concentrations reduced the motility of *Campylobacter jejuni* as well as interfered with quorum-sensing activity and biofilm formation (Castillo et al., 2015). A similar effect was observed when lactic acid was applied at a concentration of 0.6% in *Salmonella typhimurium* (Burt et al., 2016). Mith et al. (2015) also found that oregano essential oil and carvacrol are able to significantly down-regulate genes involved in motility of *E. coli* O157:H7 (Mith et al., 2015). Therefore, since the antimicrobial is a mixture of organic acids and plant extracts (e.g., oregano), it is likely that the reduced motility observed in *C. jejuni* and *C. coli* is a result of the down-regulation of genes involved in motility.

Motility is an important virulence factor in *Campylobacter jejuni* (Corcionivoschi et al., 2012) that can be affected by structural and quantitative changes in surface polysaccharides (CPS). These changes are very important because similar to other bacterial pathogens, *Campylobacter* spp. expresses capsular polysaccharides as a virulence factor to avoid opsonophagocytosis (Nanra et al., 2013). CPS was also previously shown to play an important role in systemic infections as well as in induction of ovine abortion due to antigenic variation and immune evasion (Sahin et al., 2017). Interestingly, our study shows that changes were only detected for *C. jejuni* RC039 CPS profiles but absent in *C. coli* RC013. This is possibly caused by the significant diversity in gene content between *C. jejuni* and *C. coli* (Dorrell et al., 2001). These variations for both CPS and LOS could be reflected in variable effects on the bacterial virulence potential.

At gene expression level, it was described previously that sub-inhibitory concentrations of antimicrobials influences gene transcription levels in many bacterial pathogens (Tsui et al., 2004; Donoghue et al., 2015; Oh and Jeon, 2015b). Since the sub-inhibitory concentrations of Auranta 3001 did not inhibit the growth of *C. jejuni* RC039 and *C. coli* RC013, the reduction, observed in the virulence attributes, could be due to the effect of the antimicrobial on the transcription of the virulence genes. Therefore, we used qRT-PCR to determine the effect of Auranta 3001 on the expression of *cetB* and *hcp* genes of *Campylobacter*. *CetB* is involved in energy chemotaxis and has been described to play a role in motility (Konkel et al., 1997; Reuter et al., 2010; Hermans et al., 2011a). The *hcp* gene encodes for a hemolysin-correlated protein and is a key indicator for a functional T6SS (Corcionivoschi et al., 2015; Ugarte-Ruiz et al., 2015). It has been shown that inactivation of *C. jejuni* T6SS resulted in a reduction of adherence to and invasion of *in vitro* cell lines, while over-expression of a hemolysin co-regulated protein (*hcp*), greatly enhanced these processes (Lertpiriyapong et al., 2012). During our study, we observed that Auranta 3001 modulated the transcription level of genes coding energy chemotaxis (*cetB*) and hemolysin correlated protein (*hcp*) thus indicating that the anti-*Campylobacter* colonization effect observed with Auranta 3001 could be mediated via down-regulation of critical colonization genes.

Chicken broilers are still the main source of *Campylobacter*-related infections. Development of novel strategies and products that could be efficient in reducing the colonization levels in poultry is currently becoming a priority for the industry (Johnson et al., 2017). Our *in vivo* results indicate a reduction in colonization up to 6 logs between treated and untreated groups. These results clearly show that the anti-pathogenic effect of Auranta 3001, observed *in vitro*, also translates in less colonization *in vivo*. In conclusion, our results indicate that Auranta 3001 is able to significantly reduce the invasiveness of *C. jejuni* and *C. coli* and protect the host cells *in vitro* and *in vivo* against *Campylobacter* infection and colonization. This effect is expressed by its negative impact on important virulence factors such as motility, adherence, and internalization. Based on these results, Auranta 3001 shows great potential as a method to control campylobacteriosis and provides more information in regards to the mechanistic mode of action of novel antimicrobial products and extracts. Further *in vivo* studies could potentially explain the mechanism of action, by characterizing the genomic, transcriptomic, and proteomic profiles of the pathogens.

## Author Contributions

FS, AS, CK, ML, LP, and LS conceived the design and performed the experiments. FS, AS, NC, LS, and VL analyzed the data. NC, PW, and LS contributed reagents, materials, and analysis tools. NC, VL, AS, FS, and OG wrote the paper. All authors read and approved the final manuscript.

## Conflict of Interest Statement

The authors declare that the research was conducted in the absence of any commercial or financial relationships that could be construed as a potential conflict of interest.
