# Supplementary material for: A Novel Natural Antimicrobial Can Reduce the in vitro and in vivo Pathogenicity of T6SS Positive Campylobacter jejuni and Campylobacter coli Chicken Isolates
Source: Front Microbiol. 2018 Sep 7;9:2139. doi: 10.3389/fmicb.2018.02139 (PMC6137164; doi:10.3389/fmicb.2018.02139)
Supplement: Supplementary file 1 [file Table_1.DOCX]

**Supplementary figure 1.** Adhesion and invasion of *C. jejuni* RC039 to HCT-8 cells. Pre-infection treatment of HCT-8 cells with 0.1% (panel A) and 0.5% (panel B) Auranta 3001 also leads to reduced internalisation. If bacteria only are exposed to 0.1% (panel C) and 0.5% (panel D) of Auranta 3001 also affects internalisation. Results are expressed as percentages of the initial inoculum. Asterisks indicate significant differences (Student’s *t* test *p<0.05; **p<0.01; ***p<0.001). Error bars represent the standard deviation of means from three different experiments, each containing triplicate samples.

**Supplementary figure 2.** Adhesion and invasion of *C. coli* RC013 to HCT-8 cells. Pre-infection treatment of HCT-8 cells with 0.1% (panel A) and 0.5% (panel B) Auranta 3001 also leads to reduced internalisation. If bacteria only are exposed to 0.1% (panel C) and 0.5% (panel D) of Auranta 3001 it also affects internalisation. Results are expressed as percentages of the initial inoculum. Asterisks indicate significant differences (Student’s *t* test *p<0.05; **p<0.01; ***p<0.001). Error bars represent the standard deviation of means from three different experiments, each containing triplicate samples.
