# Supplementary figures and images for: A Novel Natural Antimicrobial Can Reduce the in vitro and in vivo Pathogenicity of T6SS Positive Campylobacter jejuni and Campylobacter coli Chicken Isolates
Source: Front Microbiol. 2018 Sep 7;9:2139. doi: 10.3389/fmicb.2018.02139 (PMC6137164; doi:10.3389/fmicb.2018.02139)

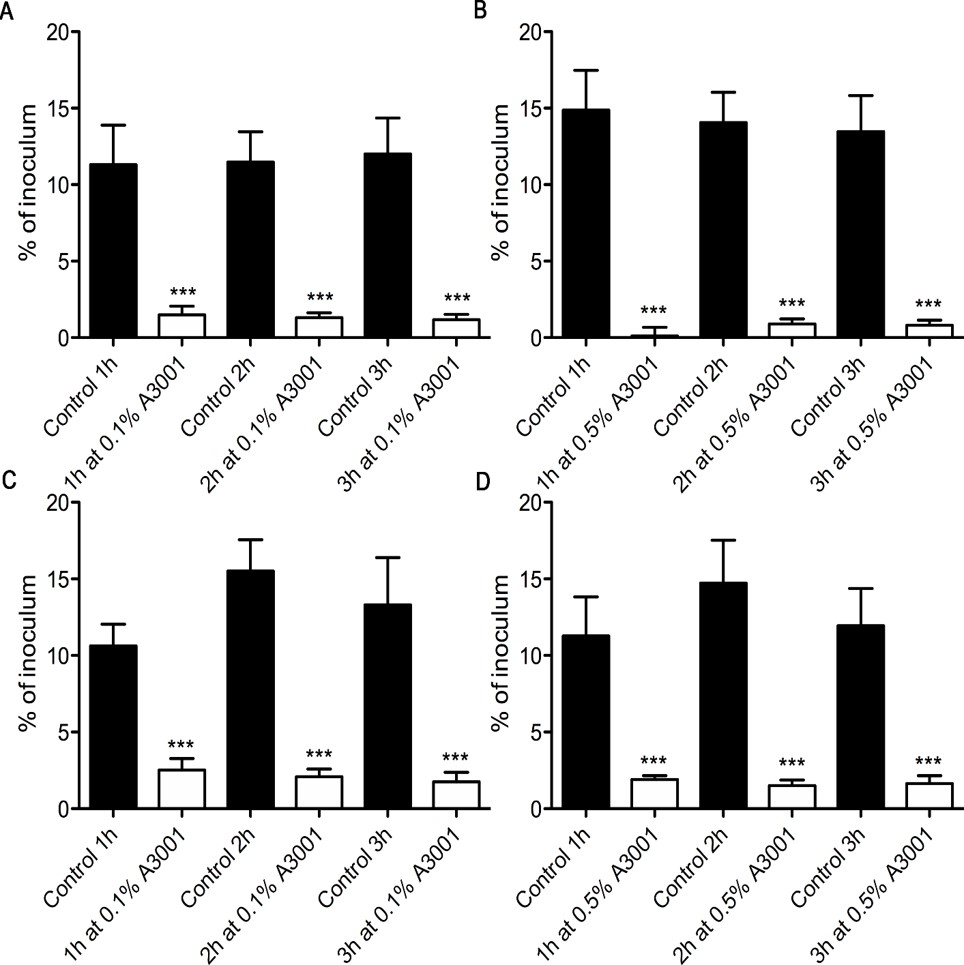

Supplement: Supplementary file 2 [file Image_1.JPEG]

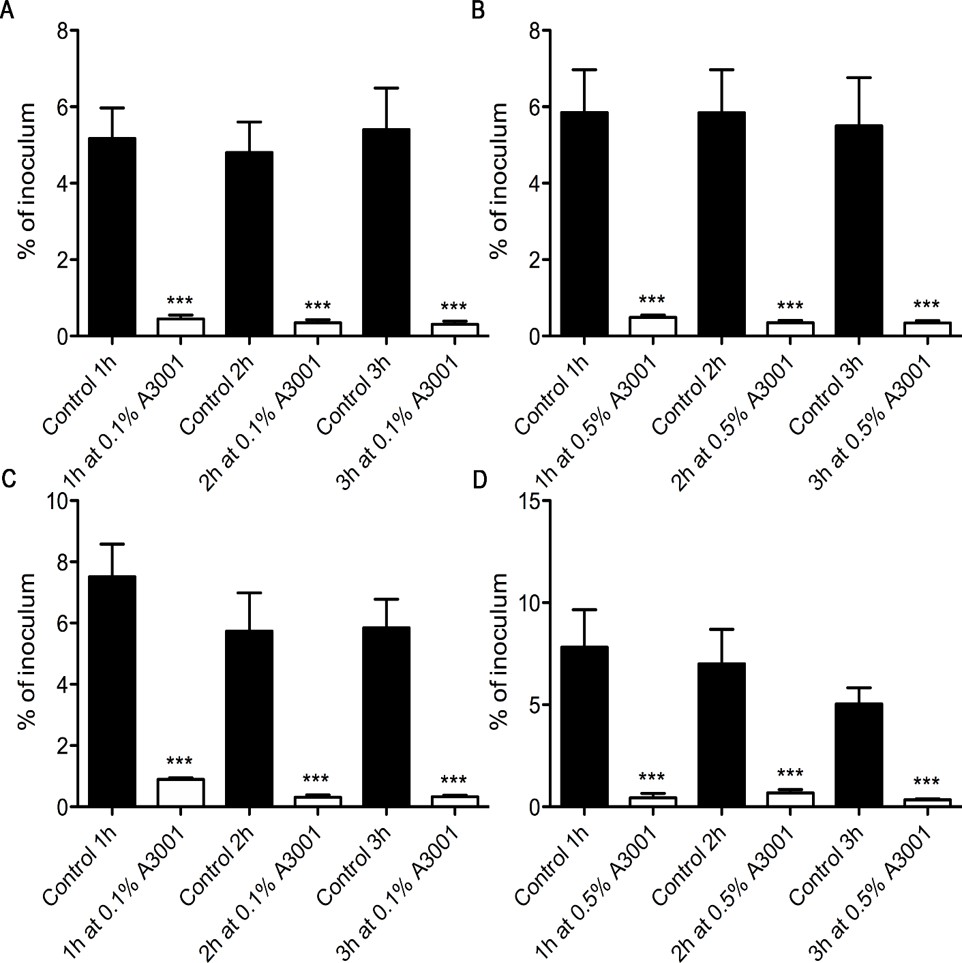

Supplement: Supplementary file 3 [file Image_2.JPEG]
